# Physics of Ice Nucleation and Antinucleation: Action of Ice-Binding Proteins

**DOI:** 10.3390/biom14010054

**Published:** 2023-12-30

**Authors:** Bogdan S. Melnik, Ksenia A. Glukhova, Evgeniya A. Sokolova (Voronova), Irina V. Balalaeva, Sergiy O. Garbuzynskiy, Alexei V. Finkelstein

**Affiliations:** 1 Institute of Protein Research, Russian Academy of Sciences, 142290 Pushchino, Russia; gkseniya@gmail.com (K.A.G.); sergey@phys.protres.ru (S.O.G.); 2 Institute of Biology and Biomedicine, Lobachevsky State University of Nizhny Novgorod, 603022 Nizhny Novgorod, Russiairin-b@mail.ru (I.V.B.); 3 Faculty of Biotechnology, Lomonosov Moscow State University, 142290 Pushchino, Russia; 4 Faculty of Biology, Lomonosov Moscow State University, 119192 Moscow, Russia

**Keywords:** ice nucleation, freezing, melting, ice-binding proteins, antifreeze proteins, ice nucleators, supercooling, ice-binding surfaces

## Abstract

Ice-binding proteins are crucial for the adaptation of various organisms to low temperatures. Some of these, called antifreeze proteins, are usually thought to inhibit growth and/or recrystallization of ice crystals. However, prior to these events, ice must somehow appear in the organism, either coming from outside or forming inside it through the nucleation process. Unlike most other works, our paper is focused on ice nucleation and not on the behavior of the already-nucleated ice, its growth, etc. The nucleation kinetics is studied both theoretically and experimentally. In the theoretical section, special attention is paid to surfaces that bind ice stronger than water and thus can be “ice nucleators”, potent or relatively weak; but without them, ice cannot be nucleated in any way in calm water at temperatures above −30 °C. For experimental studies, we used: (i) the ice-binding protein mIBP83, which is a previously constructed mutant of a spruce budworm *Choristoneura fumiferana* antifreeze protein, and (ii) a hyperactive ice-binding antifreeze protein, RmAFP1, from a longhorn beetle *Rhagium mordax*. We have shown that RmAFP1 (but not mIBP83) definitely decreased the ice nucleation temperature of water in test tubes (where ice originates at much higher temperatures than in bulk water and thus the process is affected by some ice-nucleating surfaces) and, most importantly, that both of the studied ice-binding proteins significantly decreased the ice nucleation temperature that had been significantly raised in the presence of potent ice nucleators (CuO powder and ice-nucleating bacteria *Pseudomonas syringae*). Additional experiments on human cells have shown that mIBP83 is concentrated in some cell regions of the cooled cells. Thus, the ice-binding protein interacts not only with ice, but also with other sites that act or potentially may act as ice nucleators. Such ice-preventing interaction may be the crucial biological task of ice-binding proteins.

## 1. Introduction

Many organisms on Earth must deal with temperatures below 0 °C, and hence with the potentially hazardous process of water freezing.

To control the formation of ice, the organisms use different substances varying from low-molecular ones, such as polyols and sugars [1,2], to macromolecules like ice-binding proteins (IBPs); for many of them, called antifreeze proteins (AFPs), an antifreeze activity has been demonstrated (for review, see, e.g., [3,4,5]). AFPs are perhaps the most interesting case, because their antifreeze effect requires a 200–500 times lower molecular concentration than that of low-molecular substances [6,7,8]. It is believed that these proteins save organisms from freezing because they inhibit the growth and/or recrystallization of ice crystals [8,9,10,11,12]. The antifreeze proteins were first found in the blood of fish living in the Arctic and Antarctic waters [13,14]. Then, various IBPs—and AFPs among them—were found in other animals [15], including insects [6,16,17], and many other organisms, from bacteria [18,19] and other microorganisms [20,21], to fungi [18,22] and plants [23,24].

Although IBPs—and especially AFPs—are rather extensively studied, the detailed mechanism of their action is still far from being clear [25,26,27]; but it is commonly believed that AFPs act on already existing ice crystals through binding to certain planes of the crystals [9,11,12,28,29]. However, prior to this, ice must somehow appear in the organism. Except for inoculative freezing, when ice enters an organism from outside [1,30,31,32], ice can only result from nucleation within the organism. The ice nucleation is the phenomenon we consider here.

It is well-known that water per se does not start freezing at 0 °C and stays supercooled at small and moderate negative temperatures for a very long time (see, e.g., [33,34]). The emergence of an ice seed (the smallest stable piece of rising ice) can occur in bulk calm water only at temperatures below −30 °C–−40 °C [35,36], which are much lower than normal biological temperatures. The physical theory shows that, for kinetic reasons, at higher—but still negative—temperatures, some “ice nucleators” are required to initiate the process of freezing, and that any surfaces that bind ice stronger than liquid water can work as such ice nucleators [37,38,39,40,41,42,43], though maybe not the most potent ones. Various ice nucleators can be targets for “antinucleators” that inhibit ice nucleation, and some antifreeze proteins are among them [44,45].

Here, we show that ice is nucleated in calm water in plastic test tubes at −10 °C–−15 °C (i.e., at the temperatures where ice cannot emerge without some ice nucleators, which means that the walls of these test tubes or ice-binding surfaces of some dust particles existing in water are such ice nucleators). We also show that one of the two studied IBPs—but not another—significantly decreased the ice nucleation temperature of this water (and thus, it hinders the action of relatively weak ice nucleators existing in this water). Most importantly, we reveal that both of the studied IBPs definitely decreased the ice nucleation temperature that was raised up to −3 °C–−5 °C in the presence of potent ice nucleators.

Lastly, we show that living cells have regions where IBPs concentrate at a temperature close to 0 °C. These regions may be able to act as ice nucleators, but it is unlikely that they have evolved as such, because these were human cells.

## 2. Results

### 2.1. Ice Nucleation and Its Hindering in the Presence of Ice-Binding Proteins: An Experimental Study

We studied the action of two ice-binding proteins on the temperature needed for the initiation of ice formation in the presence and absence of potent ice nucleators.

The first ice-binding protein used in our experiments was mIBP83 [46], a mutant of the natural ice-binding protein cfAFP, isoform 337 [47,48,49]; cfAFP is an antifreeze protein from a spruce budworm *Choristoneura fumiferana*, a moth whose larvae spend winter at temperatures below −30 °C [50]. This mutant was used because, while retaining the ability to bind to ice [46], it is less susceptible to aggregation during isolation and purification than the wild-type cfAFP, thus being more convenient for experiments. The mutant mIBP83 has one SS bond vs. four of the wild-type cfAFP and slightly truncated N- and C-termini (for details, see [46], as well as Supplementary Materials).

The second ice-binding protein was RmAFP1 [51], which is a wild-type antifreeze protein of a longhorn beetle *Rhagium mordax*.

The antifreeze and ice-binding activities of RmAFP1 are demonstrated in [51]. Therefore, RmAFP1 is both an antifreeze protein (AFP) and an ice-binding protein (IBP). For the mutant mIBP83, the ice-binding activity was demonstrated [46] but, strictly speaking, it is not known if this mutant retains the antifreeze activity that had its wild-type original form cfAFP, isoform 337 [47]. Thus, although the antifreeze activity of mIBP83 is likely, we, for accuracy, only call this mutant an “ice-binding protein” (IBP) rather than “antifreeze protein” (AFP), but bear in mind that its wild-type form was an AFP.

**Figure 1:**
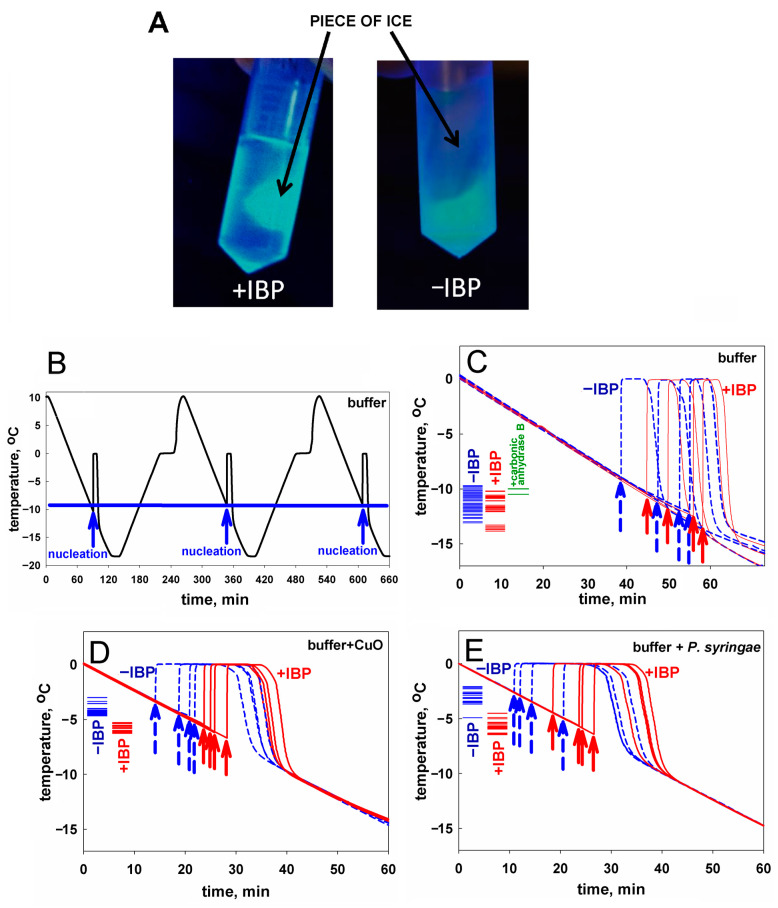
(**A**) A visualization of interaction of the mIBP83-GFP protein with ice. A comparison of two test tubes with pieces of ice in solutions: with mIBP83-GFP (+IBP) and solely with GFP (−IBP). As seen, mIBP83-GFP is bound to ice, while GFP alone (without mIBP83) is not; see also Figures S2 and S3 in Supplementary Materials as well as in [46]. (**B**–**E**) Representative examples of experiments on ice nucleation in different liquid samples in test tubes placed into a thermostat (data for the complete set of experiments are presented in Table 1). The arrows indicate the moment of ice nucleation during cooling. (**B**) An example of nucleation temperature detection in several cycles of cooling alternating with heating, for 20 mM sodium phosphate buffer, pH 7.0. The ice melting event (seen as the shoulder on the rising part of the curve corresponding to heating) was briefly discussed in [54,55]. But here, we are solely interested in the ice nucleation at cooling—see the beginnings (marked by arrows) of the sharp peaks on the falling parts of the curve. Throughout this experiment, the sample and the test tube remained unchanged, and, as seen, the nucleation temperature was practically the same (±0.4°) for all cycles. Analogous “nucleation peaks” (indicated by arrows) for different samples in different test tubes are shown separately in panels (**C**–**E**). (**C**) Testing an influence of the ice-binding protein on ice nucleation in the buffer. Four blue dashed lines with dashed arrows show cooling of the buffer without mIBP83 (−IBP); four solid red lines with solid arrows show the same buffer supplemented with 0.6 mg/mL mIBP83 (+IBP); this IBP concentration of 0.6 mg/mL is a commonly used antifreeze protein concentration (see, e.g., [56]). The columns of short lines on the left part of the panel indicate the experimental freezing temperatures found in all experiments: blue for the −IBP case, red for the +IBP case, and green for the control protein. The nucleation temperature is seen to be only approximately reproduced after changing the test tube and the liquid sample, but the nucleation temperature range is almost the same for both −IBP and +IBP cases. (**D**,**E**) Testing an influence of the ice-binding protein on ice nucleation by potent nucleators. The same experiments with the nucleators CuO and *P. syringae*, in the same buffer. The ice-binding protein mIBP83 reliably decreased the nucleation temperature. Concentrations/amounts of all substances are given in the caption of Table 1.

To visualize the results of some of our experiments (e.g., Figure 1A), we used the fusion protein mIBP83-GFP, where “GFP” means the cycle3 mutant form of the green fluorescent protein [52,53].

The fusion protein mIBP83-GFP, as well as mIBP83, RmAFP1, and GFP proteins, were expressed in *E. coli* cells, isolated, and purified (see Supplementary Materials).

To test the ice-binding ability of mIBP83 to ice, we took the mIBP83-GFP fusion protein, and the following experiment [46] was carried out. Two identical tubes were filled with buffer solution and were frozen at −20 °C, then incubated at room temperature till the beginning of ice melting. Thus, each tube had a piece of ice surrounded by liquid. Then, mIBP83-GFP solution was added into one tube, and GFP solution was added into another one. The tubes were irradiated using a transilluminator. If mIBP83, as a part of the fused protein, has ability to bind to ice, it should cover the piece of ice in the tube, and the piece should fluoresce more intensely than the solution.

The ice-binding ability of the fusion protein mIBP83-GFP and the lack of such an ability in GFP [46], which is observed in the experiment, is shown in Figure 1A. In the test tube marked “+IBP”, one can see a luminous piece of ice covered with mIBP83-GFP. In the test tube marked “−IBP”, it can be seen that the piece of ice is not glowing, which shows that it is not covered with GFP when GFP is not bound to IBP; only the liquid is glowing. For details of the experiment as well as for other photos, see Materials and Methods as well as Supplementary Materials (the “Examination of the ability of mIBP83-GFP protein to bind to ice surface” section and Figures S2 and S3).

Experiments on sample freezing using the thermostat (the device was described in detail in [54], see also Materials and Methods) showed the impact of mIBP83 on ice nucleation. The experiments were carried out as follows. In the thermostat, a plastic (polypropylene) test tube with a 1 mL sample was cooled from +10 °C to −18 °C at a rate of 0.24 °C/min and then heated at the same rate; the temperature of the center of the sample was measured. In Figure 1B, we show the change in temperature of sodium phosphate buffer without any proteins in several sequential cooling/heating cycles. During this continuous experiment, we repeatedly used one and the same sample portion and one and the same test tube. Freezing of the sample manifested itself in a sharp increase in the temperature of the sample upon cooling because the sample started to receive the latent heat released by the freezing liquid.

The beginning of each peak, i.e., the nucleation event, is indicated by an arrow. After the ice freezing is completed, the temperature drops back to the thermostat temperature. One can see that all three nucleation events shown in Figure 1B occur at a temperature of about −10 °C. These nucleation temperatures are very well reproducible from cooling to cooling, provided that both the sample and the test tube remain the same during the experiment.

Similar experiments previously carried out by two of us, with distilled water in the same experimental conditions, showed similar results [55].

In Figure 1C, four blue curves stand for the freezing of the same buffer, but with different portions of the sample liquid in different test tubes. We present an individual freezing curve for each portion of the sample liquid; the point of ice nucleation, i.e., the beginning of the temperature peak, is indicated with a dashed blue arrow. One can see that here, the range of nucleation temperatures is wider than in the case of several nucleation events observed for the same sample portion in the same test tube (Figure 1B). Four red curves with red arrows correspond to the solution of the mIBP83 protein in the same buffer. There is no significant change in the average nucleation temperature between the sole buffer and the buffer with added mIBP83 (see Figure 1C and Table 1).

Similar experiments with the same results were performed, as a control, with 0.6 mg/mL solution of carbonic anhydrase B, the protein that has never been considered as an antifreeze or ice-binding protein, in the same phosphate buffer; again, we saw no change in the nucleation temperature between the buffer and the buffer with carbonic anhydrase B (the difference is 0.9 ± 1.0°).

In contrast, in the presence of the nucleating agents CuO and *P. syringae*, we observed: (i) a significant (+7.0–+8.3°) increase in the ice nucleation temperature, and (ii) a significant decrease in the nucleation temperature upon the addition of mIBP83 in the presence of the nucleating agents (see Figure 1D,E, and Table 1). One can see, however, that the nucleation temperature in the presence of nucleators + mIBP83 is still higher than the nucleation temperature in the pure buffer (cf. Figure 1D,E, with Figure 1C). This means that the effect of the “antifreeze” IBP and both nucleators may depend on their concentrations and/or heterogeneity, which will be studied separately.

The nucleation temperatures for all studied samples are given in Table 1. Table 1 and Figure 1 show that mIBP83 decreases the ice nucleation temperature only in the presence of a potent ice-nucleating agent.

Similar experiments have been performed with a hyperactive antifreeze protein RmAFP1 from a longhorn beetle, *Rhagium mordax* [51].

**Figure 2:**
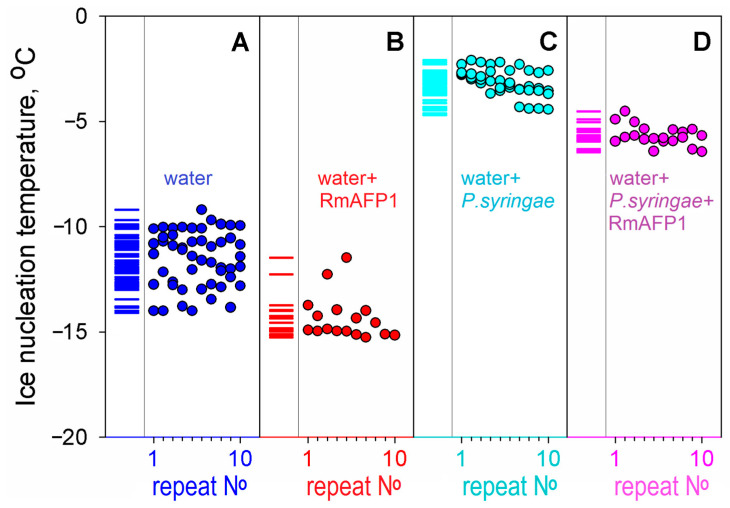
Experiments on the effect of an antifreeze protein RmAFP1 on ice nucleation. We have performed 2–5 series of experiments for each liquid (**A**–**D**); each series consisted of 10 or more repeats of a cycle of cooling, alternated with heating, without replacing the liquid sample and test tube—as in the experiment demonstrated in panel (**B**) in Figure 1. The repeat No in each of the series for each liquid is indicated at the bottom of each panel. (**A**) Water without any proteins, pH 7.0. (**B**) Water supplemented with 0.04 mg/mL RmAFP1. (**C**) Water with *P. syringae* as an ice nucleator (cf. Figure 1E). (**D**) Water with the nucleator *P. syringae* supplemented with an antifreeze protein RmAFP1 (0.04 mg/mL). The repeats after No 10 are not shown in the panels for the sake of compactness, but columns of short lines on the left part of the panels indicate the experimental nucleation temperatures found in all repeats of all experiments. The figure shows that, although the antifreeze protein RmAFP1 altered the ice nucleation temperatures in the absence of *P. syringae* ice nucleator, the impact of RmAFP1 is even more pronounced in the presence of the ice nucleator, because the ± deviations are twice smaller in the latter case.

The results are shown in Figure 2 and Table 1. It is seen that RmAFP1 decreased the ice nucleation temperature in the buffer in vitro and, as well as mIBP83, hinders the impact of potent ice nucleators.

It follows from Figure 1 and Figure 2 and Table 1 that the freezing of all studied solutions occurs not at 0 °C but, in the absence of nucleators, below −8 °C. By the way, this means that in the absence of nucleators (including pieces of ice [32] that may enter the body from outside [30]), the blood freezing per se cannot occur in any polar fish since the ocean temperature is never below −2 °C [57] (see also [58]).

In all the above cases, the initiation of freezing occurred in supercooled liquids. The phenomenon of liquid supercooling before freezing is well known [38,39]. Below, it is discussed in association with the ice nucleation kinetics. To elucidate the mechanism of freezing initiation and especially the functioning of ice-binding proteins, i.e., antifreeze proteins and ice nucleators, in Section 3, we address the theory of the first-order phase transitions [38,39,40,41] describing the nucleation of crystals, e.g., ice. We use this theory to evaluate the rate of ice formation at different temperatures in water and in bodily fluids, and, in particular, at “biological” temperatures.

We focus on the nucleation which is a crucial step of ice formation (because “there is no pregnancy without conception”) and pay almost no attention to the growth of ice, which, at “biological” temperatures, usually takes much less time than the ice nucleation event [43].

### 2.2. In Living Organisms, Can an Antifreeze Protein Bind to Something That Did Not Evolve to Be an Ice Nucleator?

Since the activity of the ice-binding proteins clearly manifests itself in the blocking of ice nucleators (see Figure 1 and Table 1), we hypothesized that antifreeze proteins could evolve to bind to any surfaces that are or may serve as ice nucleators, which potentially could be hazardous for an organism.

It is known that ice nucleators are used for quite different purposes in various organisms. Some ice nucleators (e.g., in *P. syringae*) are thought to be used as a weapon of parasitic bacteria against their hosts [59] or, in some plants, as a key constituent of a natural thermostat utilizing, in frost, the latent heat released during the nucleator-induced freezing to save other parts of the plant from freezing [60]. But one cannot expect ice nucleators to evolve in warm-blooded animals, e.g., in mice, although it has already been shown [61] that ice arises in tails of mice at −22 °C (while ice cannot appear at temperatures higher than −35 °C without nucleators (see Section 3 below)), and that an antifreeze protein induced by transfection protects the mice tails from frostbite damage. Thus, the observed ice-nucleating activity in mice is apparently an incidental side effect of something with another function.

In this regard, we checked if cells of a warm-blooded animal, i.e., human cells, happen to have binding sites for mIBP83.

Since mIBP83-GFP allows the visualization of the mIBP83 location, we transfected human breast cancer cells SKBR-3 via plasmids, encoding either the fused protein mIBP83-GFP or sole GFP as a control. The transfected cells were cultured under standard conditions (see Section 5.4 in Materials and Methods).

To test the response of the transfected cells to cold, they were kept at +37 °C and then incubated at +2 °C for 2 h, followed by immediate fixation with 4% formaldehyde to prevent protein redistribution during the imaging procedure. The temperature of +2 °C was chosen as the lowest temperature at which the cells remained spread out and attached to the substrate, and accordingly, it was convenient to use a laser scanning microscope (see Section 5).

**Figure 3:**
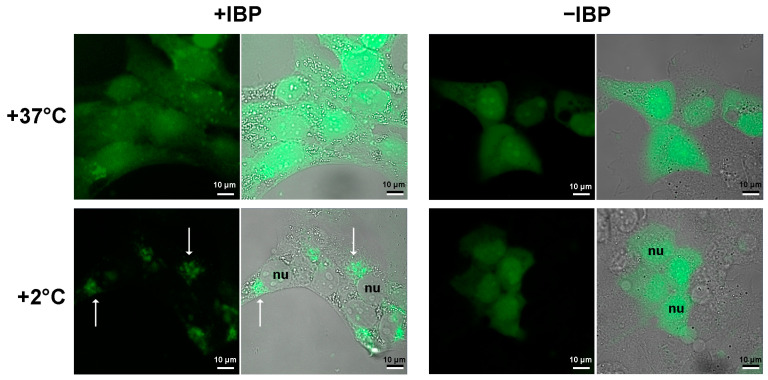
Localization of the fused protein mIBP83-GFP (+IBP) and GFP alone (−IBP) in SKBR-3 cells. The cells were kept at +37 °C or incubated at +2 °C for 2 h, then fixed and imaged using a laser scanning microscope. The fluorescence images (black background) and the merged “transmittance + fluorescence” images (gray background) are presented for each experiment. The nuclei of some individual cells are marked as nu. The white arrows indicate some of the most pronounced mIBP83-GFP accumulations in some regions of the cooled cells. It is seen that the well-defined accumulation of mIBP83-GFP (and not GFP alone) is only observed at a temperature close to 0 °C, while at +37 °C, both proteins do not accumulate in any small area in the cell.

The pattern of the intracellular location of mIBP83-GFP clearly differs from that of the sole GFP, namely at a low positive (+2 °C) temperature (Figure 3). At +37 °C, both proteins do not show a clear location in the cell. The cooling down to +2 °C leads to drastic changes in the distribution of mIBP83-GFP, but not GFP. The amount of diffusely distributed mIBP83-GFP decreases, and it accumulates mainly in some regions of the cytoplasm, including a part of the perinuclear regions. Although it is improbable that some regions in the considered cells evolved as a natural target for the given protein, mIBP83-GFP is concentrated in small areas that are clearly visible in the cells upon cooling down to almost zero.

## 3. Ice Nucleation: A Theoretical Consideration

We consider the ice nucleation at high subzero temperatures that are most interesting for biology, i.e., just below 0 °C (=273 K). Here, the ice and the liquid water phases are close to the equilibrium, and we ignore shock waves which are rare in organisms but, in principle, can trigger freezing in supercooled liquids [37].

As reported previously [38,39,42,43,55,62], the “3-dimensional case” of ice nucleation—nucleation within a body of bulk water—can only occur, for kinetic reasons, at rather low temperatures (experimentally: below ≈−35 °C [63]), which are not of interest here.

Therefore, we consider the most “biology-related” case of ice formation that occurs at high subzero temperatures on the surfaces that are in contact with water. The basic estimates of the nucleation time of this “2-dimensional case” of the first-order phase transition can be obtained using the classical theory of nucleation [40,64,65,66]. To do so, one must find the activation free energy corresponding to the transition state, i.e., the maximum value $G_{d}^{\#}$ of the free energy $G_{d}\left(n\right)$ that changes with growing *n*, the number of particles in the *d*-dimensional (*d* = 3 or 2) piece of the new phase:

(1) $$G_{d}\left(n\right)\approx n∆\mu +a_{d}n^{1-1/d}B_{d}\;,$$

where ∆μ≤0 is the chemical potential of a molecule in the “new” (arising) solid phase minus that in the “old” (liquid) one (so that ∆μ=0 at the point of thermodynamic equilibrium of phases); $B_{d}>0$ is the additional free energy of one molecule on the border of the “new” phase, i.e., on its surface for the 3-dimensional (d=3) or perimeter for the 2-dimensional (d=2) case; and $a_{d}n^{1-1/d}$ (where $a_{d=2}\approx (1.77÷2)d$, $a_{d=3}\approx (1.6÷2)d$; see [43]) is the number of molecules on the border of a compact piece of the new phase of n≫1 particles. Then, $G_{d=3}^{\#}={\frac{a_{3}B_{3}}{3}\left({\frac{2}{3}a}_{3}\frac{B_{3}}{-∆\mu }\right)}^{2}$ and $G_{d=2}^{\#}=\frac{{\left(a_{2}B_{2}\right)}^{2}}{4(-∆\mu )}$, while the diameter of the ice “seed” (i.e., the minimal stable piece of arising ice) is

(2) $$D_{seed}\approx 3Å\cdot \frac{a_{d}B_{d}}{-∆\mu }$$

in both cases [43], with 3Å being the size of an H_2_O molecule.

The value of the temperature-dependent term ∆μ is estimated as follows. At the temperature $T_{0}-∆T$ ($T_{0}=2$73 K, i.e., 0 °C, is the water/ice equilibrium point, and $0\leq ∆T{\ll T}_{0}$), $∆\mu =-∆S_{\left(1\right)}\cdot (-∆T)\equiv -∆H_{\left(1\right)}\left(\frac{-∆T}{T_{0}}\right)$ according to classical thermodynamics, where $∆S_{\left(1\right)}$ and $∆H_{\left(1\right)}$ are the entropy and enthalpy of water freezing per 1 molecule at the absolute temperature ${T=T}_{0}$. Taking $∆S_{\left(1\right)}$ and $∆H_{\left(1\right)}$ values from [67], we obtain [43]:

(3) $$\frac{∆\mu }{k_{B}T_{0}}\approx \frac{-∆T}{100{}^{\circ}},$$

where $k_{B}$ is the Boltzmann constant. Thus,

(4) $$D_{seed}\approx 3Å\cdot a_{d}(B_{d}/k_{B}T_{0})\cdot \frac{100{}^{\circ}}{∆T};$$

with the value $B_{d}\;$≈ 0.85$k_{B}T_{0}$ that follows from the experimental value of the ice/water interface free energy ≈32 erg/cm^2^ [68] and the fact that an H_2_O molecule occupies ≈10 Å^2^ of the interface, we obtain

(5) $$D_{seed}\approx \frac{1300{}^{\circ}}{∆T}\;Å.$$

The time of appearance of the ice seed around *one given* H_2_O molecule is

(6) $$t_{1,d}\sim \;\tau \cdot exp\left(\frac{{+G}_{d}^{\#}}{k_{B}T}\right),$$

where *τ* (the time of the border H_2_O molecule diffusive inclusion in or exclusion from the ice surface at about 0 °C) is a fraction of a microsecond [39,43]. It is clear that $exp\left(\frac{G_{d}^{\#}}{k_{B}T}\right)$ is the main temperature-dependent term here (when ∆T→0 and thus ∆μ→0, i.e., close to 0 °C, $G_{d}^{\#}$ can be huge), while the temperature dependence of the term τ is relatively weak [43] and can be ignored.

The time of nucleation, i.e., of the appearance of an ice seed, around *one of the N* water molecules contained in (at *d* = 3) the vessel or on its borders (at *d* = 2) is

(7) $$t_{N,d}^{\left(1\;seed\right)}\sim \;t_{1,d}/N,$$

and $t_{N,d}^{\left(1\;seed\right)}$ is much larger than the time of ice growth after the seeding, especially close to 0 °C. Both theoretically and experimentally, the growth of ice in a ~1 mL test tube at ≈−10 °C usually takes seconds, while the ice nucleation time ($t_{N,d}^{\left(1\;seed\right)}$) at temperatures higher than −10 °C is usually in minutes, hours, or much more [39,43,55].

Note that if, as observed experimentally, the time of ice appearance in a test tube, $t_{N,d}^{\left(1\;seed\right)}$, is much longer than 10 s, and $N\sim 10^{15},$ which corresponds to the volume of a tiny droplet or the water layer on walls of a ~1 mL test tube, then $t_{1,d}$, the appearance of the ice seed around *one given* H_2_O molecule, takes *billions* of years, like the decay of a uranium nucleus. A comparison of this $t_{1,d}≳10^{9}$ years with the experimental times of ice nucleation in a ~1 mL test tube ($t_{N,d}^{\left(1\;seed\right)}\sim 40$ s at the temperature of ice nucleation; see the end of this section and Section 3.2 below) and the subsequent ice growth time (also ~10 s; see [55]) shows that all ice in a ~1 mL test tube usually arises from one or two, and rarely three, ice seeds.

If the time of appearance of the ice seed around one given H_2_O molecule is $t_{1,d}$, the probability that a seed *does not* appear around the given H_2_O molecule in time *t* is $exp\left(-t/t_{1,d}\right)$, and the probability that a seed arises around this H_2_O molecule is $1-exp\left(-t/t_{1,d}\right)\approx t/t_{1,d}$ if $t/t_{1,d}\ll 1$. Under the condition that $t/t_{1,d}\ll 1$, the probability of the appearance of *m* seeds in time *t* in an ensemble of *N* water molecules follows from the Poisson probability distribution $\mathrm{Prob}\left(m,N,t/t_{1,d}\right)$ = $\frac{{\left(Nt/t_{1,d}\right)}^{m}exp\left(-Nt/t_{1,d}\right)}{m!}$, which gives the average expected value of *m* as $\bar{m}=Nt/t_{1,d}$, and its variance as $\bar{{\left(\delta m\right)}^{2}}=Nt/t_{1,d}$. Thus, the expected value of *m* is $Nt/t_{1,d}\pm \sqrt{Nt/t_{1,d}}$. Therefore, at $\bar{m}=1$, 1±1 is the range of expected seed numbers at the characteristic moment $t=t_{1,d}/N{\approx t}_{N,d}^{\left(1\;seed\right)}$ (see Equation (7)) of the appearance of the first ice seed in the ensemble. This means that the expected characteristic time range of appearance of the first ice seed at a fixed temperature is approximately $t_{N,d}^{\left(1\;seed\right)}\pm {\;t}_{N,d}^{\left(1\;seed\right)}$.

### 3.1. Ice Nucleation in Bulk Water Is Only Possible at Rather Low Temperatures

For the 3-dimensional case corresponding to the ice nucleation in a body of bulk water, the transition-state free energy is:

(8) $$G_{d=3}^{\#}={\frac{a_{3}B_{3}}{3}\left({\frac{2}{3}a}_{3}\frac{B_{3}}{-∆\mu }\right)}^{2}\approx {14k_{B}T_{0}\left(\frac{100{}^{\circ}}{∆T}\right)}^{2},$$

where $B_{3}\;$≈ 0.85$k_{B}T_{0}$ [43] (see above).

Equations (6) and (8) show that the time of ice appearance is extremely temperature sensitive: it turns to infinity when ∆T→0, and, unlike most molecular processes, the freezing is accelerated not with increasing but with decreasing temperature, at least when it is not too far from 0 °C.

The time of ice appearance within 1 mL of resting pure water containing $N\approx 3\cdot 10^{22}$ H_2_O molecules not surrounded by solid walls (e.g., inside a water droplet) should take (theoretically) many years at about −35 °C, and a fraction of a microsecond at about −50 °C [43]; this is in agreement with numerous experimental observations that ice never appears within a droplet of resting pure water at −33 °C and above [36].

### 3.2. Ice Nucleation on the Ice-Binding Surfaces at High Subzero Temperatures

We herein address a more biologically interesting case of ice formation on (potentially) ice-binding surfaces that interact with ice stronger than with liquid water, that is, which binds water molecules in any configuration suitable for ice formation. These surfaces can be potentially ice-binding walls of vessels or surfaces of ice-binding dust particles. Unlike the ice nucleation inside a body of bulk water, the ice nucleation on a surface can occur at rather high subzero temperatures [38,39,41,43].

**Figure 4:**
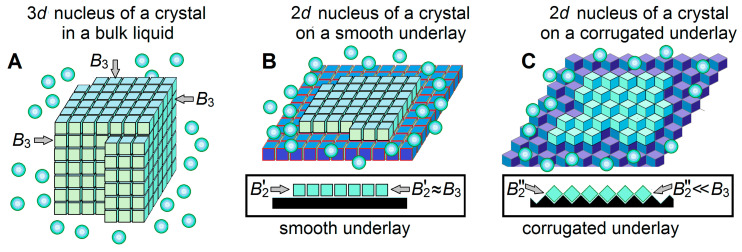
Schematic drawings of a 3-dimensional (3D) ice nucleus (**A**), and two kinds (**B**,**C**) of 2-dimensional (2D) ice nuclei on underlays of different shapes. The water molecules in ice are shown as light-blue cubes, the surrounding liquid water molecules are shown as light-blue balls, and ice-binding surfaces (underlays) are shown in dark-blue or black. Additional free energies $B_{3}$ of molecules on different facets of the 3D ice nucleus, in principle, may be somewhat different, since these molecules may have different orientations relative to different facets [39,69]. The 2D nuclei arise on the underlying ice-binding (or ice) surfaces. In extreme cases, the underlays may be smooth (**B**) or corrugated (**C**); side views (see insets) show that contacts between the ice molecules inside a layer formed on a smooth underlay are strong, while contacts between the ice molecules inside a layer formed on a corrugated underlay are weak, while the contact of this ice layer with the underlay is stronger in case (**C**) than in case (**B**). Respectively, the additional free energy of a border molecule of the layer arising on a smooth underlay $\left(B_{2}^{\prime }\right)$ is high, while the additional free energy of a border molecule of the layer arising on a corrugated underlay $\left(B_{2}^{\prime \prime }\right)$ is low. Thus, ice nucleation time drastically decreases on corrugated surfaces as compared to smooth ones.

On the ice-binding surface, an ice nucleus (and seed) arises not as a 3D (Figure 4A) but as a 2D (Figure 4B,C) object. This (cf. Equation (8) with Equation (9) below) drastically decreases [43] the transition-state free energy when ∆T→0:

(9) $$\frac{G_{d=2}^{\#}}{k_{B}T_{0}}=\frac{{\left(a_{2}B_{2}\right)}^{2}}{4(-∆\mu )}\;\approx 400{}^{\circ}\frac{{\left(B_{2}/k_{B}T_{0}\right)}^{2}}{∆T}\;.$$

If it is assumed that $B_{2}\approx B_{3}\approx 0.85{\;k}_{B}T_{0}$ for a 2D nucleus, as it is for the 3D one, then $\frac{G_{d=2}^{\#}}{k_{B}T_{0}}\approx \frac{300{}^{\circ}}{∆T}$, and, according to Equations (6) and (7), the characteristic time of appearance of an ice seed somewhere on the 1 mL vessel walls accommodating $N_{S}\sim$10^15^ water molecules is

(10) $$t_{N_{S},d=2}(∆T)\;\sim \;\frac{\tau }{N_{S}}exp\left(\frac{G_{d=2}^{\#}}{k_{B}T_{0}}\right)=\frac{\tau }{N_{S}}exp\left(\frac{A_{2}}{∆T}\right)\sim \;\frac{10^{-7}s}{10^{15}}\cdot exp\left(\frac{300{}^{\circ}}{∆T}\right),$$

where $\frac{\tau }{N_{S}}\sim \frac{10^{-7}s}{10^{15}}$ and, at $B_{2}\approx 0.85k_{B}T_{0}$,

(11) $$\frac{A_{2}}{∆T}=400^{{}^{\circ}}\frac{{\left(B_{2}/k_{B}T_{0}\right)}^{2}}{∆T}\;\approx \frac{300{}^{\circ}}{∆T}.$$

This means that with $B_{2}=B_{3}\approx 0.85{\;k}_{B}T_{0}$, the freezing of water in a 1 mL vessel should, theoretically, take a second at ∆T ≈ 6°, that is, at a temperature of −6 °C, and a minute at −5.5 °C. Thus, any ice-binding surface can be considered as a kind of ice nucleator. The time $t_{N_{S},d=2}$ is highly temperature sensitive: at a temperature of 1° higher than −6 °C, the appearance of an ice seed would take hours, while at a temperature of 1° lower than −6 °C, it would take a millisecond.

However, the experimentally measured [68] value $B_{3}\approx 0.85k_{B}T_{0}$ represents the average free energy of the ice/water interface per interface molecule, while different facets of an ice crystal may have somewhat different values of this interface free energy due to different orientations of molecules relative to different crystal facets [39,69]. Then, if, for instance, $B_{2}\approx 1.1k_{B}T_{0}$, we have $\approx \frac{500{}^{\circ}}{∆T}$ instead of $\frac{300{}^{\circ}}{∆T}$ in Equation (10), and theoretically, the initiation of water freezing in a 1 mL vessel should take seconds at about −10 °C, and minutes at about −9 °C (the freezing initiation temperature of −9 ÷ −10 °C was observed in our experiments; see Figure 1B). With $B_{2}\approx 1.1{\;k}_{B}T_{0}$, Equation (10) has the form

(12) $$t_{N_{S},d=2}(∆T)\;\sim \;\frac{10^{-7}s}{10^{15}}\cdot exp\left(\frac{500{}^{\circ}}{∆T}\right).$$

The value of $t_{N_{S},d=2}(∆T)\;$can be experimentally measured at a given fixed temperature ${T=T}_{0}-∆T$. However, our experiments on water cooling use a constant decrease in temperature with time *t*, where $∆T\left(t=0\right)=0$ and $∆T\left(t>0\right)=\gamma \cdot t$ with γ=0.24°/min≡0.004°/s (see Section 2.1). Therefore, the total time from the beginning of the experiment to the appearance of an ice seed at a temperature of $T_{0}-∆T$ can be calculated as $\frac{∆T}{\gamma }+t_{N_{S},d=2}(∆T)$. The minimum of this calculated time must correspond to the experimental value of ∆T.

The first derivative of $\frac{∆T}{\gamma }+t_{N_{S},d=2}\left(∆T\right)$ with respect to ∆T equals to $\frac{1}{\gamma }-\frac{\tau }{N_{S}}exp\left(\frac{A_{2}}{∆T}\right)\times \left(\frac{A_{2}}{{∆T}^{2}}\right)$, which must be equal to zero at the extremum of $\frac{∆T}{\gamma }+t_{N_{S},d=2}\left(∆T\right)$. With $A_{2}\approx 500{}^{\circ}$, this extremum corresponding just to ∆T=9.2° is the minimum because the second derivative of $∆T/\gamma +t_{N_{S},d=2}(∆T)$ with respect to ∆T is positive. At ∆T=9.2°, the optimal time of freezing nucleation calculated from Equation (11) is about 40 s.

### 3.3. Ice-Binding Surfaces

As mentioned above, the emergence of ice is catalyzed by ice-binding surfaces, i.e., the surfaces that bind ice stronger than liquid water.

These can be special substances like CuO or AgI powders, or specially evolved proteinaceous complexes in bacteria like *P. syringae* (which are potent ice nucleators), or plastic test tube walls, or some in-water dust particles (which seem to be not as potent).

However, the catalytic effect is not affected by the strength of ice binding to the “non-ice” underlay, so far as this binding is stronger than the binding of liquid water. This is because the second and all further layers of ice form on the ice which is already bound to the “non-ice” underlay, and, if the ice strongly binds to the “non-ice” underlay, a monomolecular ice layer exists even at temperatures > 0 °C; however, a massive ice formation, our sole interest, can occur on this icy underlay only at temperatures below 0 °C.

Thus, any ice-binding surface, including that of a plastic test tube or some dust particles, serves as an ice nucleator but its catalytic effect on the ice emergence is determined solely by the temperature and the free energy of the border of the arising ice, i.e., by the *B*_2_ factor (see Equations (10) and (11)). The latter depends on the orientation of molecules forming the layer of ice arising on the underlay. A special shape of the underlay (cf. Figure 4C with Figure 4B) can significantly weaken the contacts between ice molecules inside the newly arising ice layer, and accordingly, reduce the values of the boundary $B_{2}$ factors. In turn, the smaller $B_{2}$ strongly decreases the freezing temperature, thereby drastically shortening the freezing time at a given temperature. The faster ice formation on surfaces corrugated at an atomic scale has been already experimentally observed [70]. Thus, the special atomic structure of the underlay can create a powerful “ice nucleator” in contrast to the plastic walls of the test tubes, which are “weak ice nucleators”.

If strong ice nucleators are added to water in a test tube with ice-binding walls, then there are two parallel freezing nucleation reactions: one is generated by the walls of the test tube, and the other by the added nucleators. If the initiation time of the freezing generated by the tube walls alone is $t_{N_{S\_walls},d=2}\;\sim \;\frac{\tau }{N_{S\;walls}}exp\left(\frac{G_{d=2}^{\#,walls}}{k_{B}T_{0}}\right)$, and the initiation time of the freezing generated by the added nucleators alone is $t_{N_{S\_walls},d=2}\;\sim \;\frac{\tau }{N_{S\;added}}exp\left(\frac{G_{d=2}^{\#,added}}{k_{B}T_{0}}\right)$, then the initiation time of the freezing in the test tube with added nucleators is:

(13) $$t_{N_{S_{walls+added}},d=2}\;\sim \;\tau {\cdot \left[N_{S,walls}exp\left(\frac{{-G}_{d=2}^{\#,walls}}{k_{B}T_{0}}\right)+N_{S,added}exp\left(\frac{{-G}_{d=2}^{\#,added}}{k_{B}T_{0}}\right)\right]}^{-1}.$$

Here, $N_{S,walls}$ is the number of water molecules on the tube walls; $N_{S,added}$ is the number of water molecules on the surfaces of the added nucleators; and $G_{d=2}^{\#,walls}$ and $G_{d=2}^{\#,added}$ are the activation free energies for nucleation on the tube walls and on the added nucleators, respectively. If $N_{S,added}$ is large enough and $G_{d=2}^{\#,added}$ is small enough, then the freezing time is determined mainly by the added ice nucleators.

If the antifreeze (“antinucleator”) protein is added, it reduces $N_{S,walls}$ in proportion to the antifreeze concentration and the antifreeze–wall binding constant, and it reduces $N_{S,added}$ in proportion to its concentration and the antifreeze–nucleator binding constant.

## 4. Discussion

The ice-binding properties of various surfaces, mainly of technical use, have been studied (see, e.g., [71,72] and references therein). However, we do not know much about the ice-binding properties of surfaces of biological origin, which can be targets for AFPs; thus, the identification of such surfaces and the study of their properties will be the next step in the investigation of the action of ice nucleators and their interaction with antifreeze proteins.

The results obtained in the experiments with living cells (Figure 3) are in line with our hypothesis that at temperatures of about 0 °C, cells may contain some potentially ice-nucleating surfaces to which antifreeze proteins can bind.

### 4.1. Notes on Antifreeze Protein Functions

It is worth emphasizing that our work supports a new view on the functioning of ice-binding (and specifically, antifreeze) proteins. Their tasks may not only include ice binding and preventing its further growth and recrystallization; they may also aim to bind—directly or through a thin layer of water molecules—to those cell or tissue surfaces where the ice nuclei can form, thus preventing the ice formation completely.

It is known that there are several classes of antifreeze proteins, and some antifreeze proteins bind to some facets of ice crystals and to some nucleators, while others bind to other facets and other partners [11,73,74].

Occasionally, ice particles can penetrate inside the organism through the body surface, guts, gills, etc. This has been experimentally observed for fishes, insects, turtles, and some other organisms [17,30,31,75,76]. These particles initiate the inoculative freezing process, which can also be blocked by ice-binding proteins.

Furthermore, the IBPs binding to some cell surfaces may contribute to their stabilization, thereby protecting them from hypothermic cold shock damage even at a temperature above 0 °C when there is no possibility of ice emergence; this is demonstrated, e.g., by a protection of human hepatoma cells by a fish AFP at +4 °C [77]. It has been experimentally shown that the expression of a tick antifreeze glycoprotein enhances cold tolerance in *Drosophila melanogaster* [78].

The proposed binding of IBPs to cell surfaces can explain both experimentally observed phenomena [29,79]: (i) the survival during strong (below 0 °C) cooling that could result in ice formation but was avoided due to the IBP-induced inhibition of ice crystal formation, and (ii) the tolerance of cells to the cold shock under moderate cooling to almost 0 °C by the stabilization of cell surfaces due to their binding to IBPs.

It should be noted that the prevention of ice formation and binding to cell surfaces (and, of course, the blocking of the ice itself—in case it still appears, one way or another, say, by the inoculative freezing) are not the only properties of IBPs. Since mIBP83 binds to ice (Figure 1A), it can stabilize the ice increasing the ice melting temperature [55,76]; thus, an IBP can serve not only as an antifreeze, but also as an ice-stabilizing or even ice-nucleating protein. However, following the above calculations (see Equation (5)), the diameter of an ice-nucleating surface must not be less than ~130 nm at ∆T≈1° and ~20 nm at ∆T≈6−7°. This agrees with the data that a large (164 kDa) antifreeze glycoprotein can initiate the formation of ice nuclei, and its ice nucleation ability was diminished after the removal of carbohydrates (92 kDa in total), while this removal did not noticeably alter its antifreeze activity [80]. Moreover, there is a correlation between the ice-nucleator “power” (that is, the maximal nucleation temperature) and the ice-nucleator’s size [81,82]. In general, it has been shown that the size is a good predictor of the temperature of ice nucleation by different IBPs [79,83], and one can change—and even switch—the behavior of the ice-binding molecule (or molecular complex) by changing its size [84].

### 4.2. Notes on Ice Nucleators

According to the literature, the ice nucleators known to date are very different both in their chemical nature and in their “nucleation power”. Most of them act in vitro at temperatures below −10 °C, while some induce freezing at a temperature above −4 °C. Among the most potent ice nucleators, there are inorganic substances such as the powders of famous AgI [85,86], CuO [43], powders of various organic substances, including surfaces of powders or drops of some steroids [87], long-chain alcohols [88], some amino acid crystals [89], and some macromolecules. It should be noted that some of these substances can be (possibly accidentally) ingested by living organisms and manifest their (possibly dangerous) ice-nucleating activity within them.

Furthermore, some whole biological objects like pollen [90] and bacteria [91,92] (or rather, their surfaces) can serve as ice nucleators. The bacterium *P. syringae* is an extremely potent ice nucleator that induces water freezing at temperatures up to −2 °C and even above [91].

Along with the relatively well-studied [92,93] bacterial ice nucleators which are large proteinaceous complexes situated on the bacterial membrane, somewhat less is known about the chemical nature of ice nucleators acting in other organisms [60,94]. Some insects have ice nucleators, both lipoproteins and proteins, in their hemolymph in summer, and lose these, especially the most potent ones, during the cold season [94,95]. The loss of ice nucleators is also observed in turtles, and these nucleators are probably ingested soil bacteria like *P. syringae* [96]. Ice-nucleating lipoproteins from the cranefly *Tipula trivittata* are not anchored to membranes but aggregate into long chains [97]. Long filamentous aggregates are also formed by the bacterial ice nucleators (of *P. syringae* and *P. borealis*) expressed in *Escherichia coli* [98,99]. In winter rye (*Secale cereale*) leaves, ice nucleators seem to be complexes of proteins, carbohydrates, and phospholipids [100]. It is known that membrane vesicles of *Erwinia herbicola* bacteria have ice-nucleating activity [101], which can be inhibited by an antifreeze glycoprotein, and it is hypothesized [102] that cell membranes by themselves could be ice nucleators, especially in animal cells, because they have a large fraction of cholesterol known as a good ice nucleator in a solid state [87,103]. Also, the pool of ice nucleators includes cellulose, which is the major component of plant cell walls [104], and even some (especially large) antifreeze proteins [79,80] (see the end of Section 3.1 above).

### 4.3. Ice Nucleators, and Antifreeze Proteins as Antinucleators

Although it remains impossible to directly observe the interaction between ice nucleators and antinucleating proteins, the hindering of the ice-nucleating activity unambiguously hints at a connection between them.

Some data on interactions between ice nucleators and antifreeze proteins are available in the literature. It was shown that antifreeze proteins from the larvae of a beetle *Dendroides canadensis* inhibit some, but not all, tested ice nucleators [95,105,106]. An antifreeze glycoprotein from Antarctic toothfish (*Dissostichus mawsoni*) was demonstrated to inhibit the ice-nucleating activity of membrane vesicles from the bacterium *Erwinia herbicola* [101]. A bacterium *Acinetobacter calcoaceticus* was shown to produce an anti-nucleating protein that demonstrated various specificities for various ice-nucleating bacteria and AgI [107]. Fish antifreeze protein type III was reported to inhibit the ice nucleation process by adsorbing onto the surfaces of both ice nuclei and dust particles [44]. Fish antifreeze proteins (AFP I and AFP III) and some simpler organic compounds like poly(vinyl alcohol), poly(vinyl pyrrolidone), and poly(ethylene glycol) inactivated the ice-nucleating activity of AgI [45]. A recombinant antifreeze protein derived from the perennial ryegrass plant *Lolium perenne* suppressed the ice nucleation point of ice nucleators of *P. syringae*, while a recombinant fish antifreeze protein had no such effect [108]. An evaluation of the effects of five different antifreeze proteins on the activity of bacterial ice nucleators showed that bacterial ice-nucleating proteins are inhibited by certain antifreeze proteins, while other antifreeze proteins produce no such effect [74].

Thus, it can be stated that our mIBP83 protein is not the only one with an antinucleating ability. At least some other antifreeze proteins, in addition to inhibiting ice growth and/or recrystallization, were shown to inhibit the action of ice nucleators, thus being able to completely prevent the formation of ice.

## 5. Materials and Methods

### 5.1. mIBP83, RmAFP1, GFP, and mIBP83-GFP Proteins

The construction, expression, isolation, and purification of the ice-binding protein mIBP83, GFP (which is the cycle3 mutant form of the green fluorescent protein [52]), as well as of the fusion protein mIBP83-GFP, were performed as described previously [46]. Using the same technique, the antifreeze protein RmAFP1 (whose sequence corresponded to the wild-type RmAFP1 isoform protein from the beetle *Rhagium mordax* [51]) was expressed, isolated, and purified. See also “Genetic constructs” and “Isolation and purification of mIBP83, RmAFP1 and mIBP83-GFP proteins” sections (as well as Figure S1) in Supplementary Materials.

### 5.2. Testing Ice-Binding Ability of mIBP83-GFP and GFP Proteins

Identical test tubes were filled with buffer solution (1.0 mL, 20 mM sodium phosphate buffer, pH 7.0) and frozen at −20 °C, and then incubated at room temperature till the beginning of ice melting. Then, mIBP83-GFP solution or GFP solution was added (200 μL, 2 mg/mL) to each test tube.

The test tubes were illuminated using an ecx-f20.m VILBER transilluminator (Collégien, France).

### 5.3. Freezing Experiment Equipment

Experiments on freezing were carried out using a Julabo F-25 thermostat (Julabo GmbH, Seelbach, Germany). The thermostat and measuring thermometers (thermocouples) were checked using an LT-300-N laboratory thermometer (TERMEX, Tomsk, Russia), resolution 0.01 °C, accuracy ±0.05 °C. In detail, the experimental device is described in [54].

The experiments used standard plastic (polypropylene) microcentrifuge test tubes (1.7 mL, Cat. No. 3621, Costar^®^ (Fisher Scientific, Pittsburgh, PA, USA)). The liquid volume was always 1 mL.

In experiments with added nucleators, we added either 0.5 mg of copper(II) oxide or 0.05 mL of suspension of *P. syringae* with a cell density of 0.1 optical units.

Copper(II) oxide (CuO) was obtained from Reachem (Moscow, Russia). During our experiment, this non-soluble CuO powder was at the bottom of the test tubes.

*P. syringae* cells (*Pseudomonas syringae* pv. *syringae*) were grown on medium L (yeast extract 5.0 g/L; peptone 15.0 g/L; NaCl 5.0 g/L) at +37 °C. The cells were grown in the liquid medium to a cell density of 1.0 optical units (via absorption at 600 nm), and then precipitated on a centrifuge at 6000× *g*, and washed twice with a solution of 20 mM Tris-HCl (pH 7.5). Lastly, the buffer of the same composition was added to obtain the desired cell density (0.1 optical units). The concentration of *P. syringae* cells was controlled via absorption at 600 nm.

### 5.4. Experiments with the Human Cell Culture

The human breast adenocarcinoma cells SKBR–3 (ATCC^®^ HTB 30^™^) were cultured in McCoy’s medium (PanEco, Moscow, Russia) with 10% (*v*/*v*) fetal calf serum (HyClone, Cytiva, Marlborough, MA, USA) in 5% CO_2_ at +37 °C.

For transient expression of fluorescent proteins, we used the plasmid vectors pTag-2N encoding the gene of mIBP83-GFP or sole GFP (or rather, cycle3 GFP) under the control of cytomegalovirus promoter and the gene of resistance to the antibiotic G418. The cells were transfected using the Lipofectamin 3000 transfection reagent (Invitrogen, Thermo Fisher Scientific, Waltham, MA, USA) according to the manufacturer’s instructions. The transfection was followed by cultivation in a selective G418-containing medium for several passages.

Cooling of the cells was performed using a solid-state ThermoStat Plus (Eppendorf^®^, Hamburg, Germany) with precise temperature control. The cells were cultured in Falcon^®^ (Corning, Glendale, AZ, USA) 96-well black/blear flat-bottom TC-treated imaging microplates or Eppendorf^®^ glass-bottom cell imaging dishes. To test the response to cold, the cell cultures were incubated at +2 °C for 2 h and then immediately fixed with 4% formaldehyde. The experimental temperature was +2 °C because at lower temperatures, the cells would separate from the substrate, thus becoming inconvenient for the microscopic research.

The images were obtained using an Axio Observer Z1 LSM–710 DUO NLO laser scanning microscopy system (Carl Zeiss, Oberkochen, Germany). The GFP fluorescence was excited at 488 nm and registered in a wide spectral range of 500–735 nm.

## 6. Conclusions

We show that the studied ice-binding protein, mIBP83, virtually does not affect the ice nucleation temperature in the buffer in test tubes but hinders the impact of potent ice nucleators of various chemical natures, namely CuO powder and ice-nucleating bacteria *Pseudomonas syringae*. Additional experiments on human cells show that mIBP83 is concentrated, but only in cooled cells, in some of their regions, which definitely did not evolve as the ice nucleators.

This supports a hypothesis that if a cell, a tissue, a blood vessel, etc., has ice-binding, i.e., potentially ice-nucleating surfaces—independently of their nature and evolutionary origin—then certain antinucleating molecules, including antifreeze proteins, are required to bind to these surfaces, thereby blocking their ice nucleation activity. And the surfaces of ice crystals (if these nonetheless appear—say, by inoculation) can be considered as a special case of surfaces on which ice can form, and therefore, such surfaces should also be blocked by antifreeze proteins.

Our future work implies a detailed investigation of the targets for IBP binding in various cell types and tissues. Specifically, we plan to investigate ice nucleators from cells and organisms that must avoid freezing, thereby revealing the interaction of their ice nucleators with some antinucleating proteins.

## Figures and Tables

**Table 1:** Ice nucleation temperatures for explored samples.

Sample	Number of Measurements	Nucleation Temperature, °C ± Deviation		Difference ^‡^
CuO * in the buffer	32	−4.1 ± 0.4	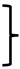	**1.7 ± 0.5**; Figure 1D
CuO * + mIBP83 ^†^ in the buffer	23	−5.8 ± 0.3		
*P. syringae* * in the buffer	21	−2.8 ± 0.5	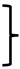	**3.0 ± 0.7**; Figure 1E
*P. syringae* * + mIBP83 ^†^ in the buffer	27	−5.8 ± 0.5		
mIBP83 ^†^ in the buffer	28	−11.9 ± 1.2	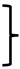	0.8 ± 1.5; Figure 1C
Buffer (sodium phosphate)	70	−11.1 ± 0.9		
			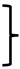	0.9 ± 1.0; Figure 1C
Carbonic anhydrase B in the buffer	2	−10.2 ± 0.4		
RmAFP1 ^†^ in water	23	−14.5 ± 1.1	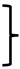	**2.9 ± 1.7**; Figure 2A,B
Water	74	−11.6 ± 1.3		
*P. syringae* * in water	53	−3.3 ± 0.7	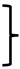	**2.5 ± 0.9**; Figure 2C,D
*P. syringae* * + RmAFP1 ^†^ in water	27	−5.8 ± 0.5		

Footnote: Concentrations/amounts per 1 mL of liquid in a polypropylene test tube: sodium phosphate buffer, 20 mM, pH 7.0; carbonic anhydrase B, 0.6 mg/mL; mIBP83, 0.6 mg/mL; CuO powder, 0.5 mg; suspension of *P. syringae*, 0.05 mL with the optical density OD = 0.1 optical units; RmAFP1, 0.04 mg/mL. ^‡^ The difference upon addition of the (potentially) antinucleating protein; the reliable differences are in bold. * Nucleator. ^†^ Antinucleator.

## Data Availability

The data presented in this study are available in this article and Supplementary Materials.

## Supplementary Materials

The following supporting information can be downloaded at: https://www.mdpi.com/article/10.3390/biom14010054/s1; Genetic constructs; Isolation and purification of mIBP83, RmAFP1, and mIBP83-GFP proteins; Examination of the ability of mIBP83-GFP protein to bind to ice surface; Figure S1: SDS-PAAG of the final stage of purification of proteins; Figure S2: Binding of mIBP83-GFP to the ice surface; Figure S3: A piece of ice in a test tube with GFP solution. References [46,52,53,109,110] are cited in the Supplementary Materials.
