# Supplementary material for: Physics of Ice Nucleation and Antinucleation: Action of Ice-Binding Proteins
Source: Biomolecules. 2023 Dec 30;14(1):54. doi: 10.3390/biom14010054 (PMC10813080; doi:10.3390/biom14010054)
Supplement: Supplementary file 1 [file biomolecules-14-00054-s001.zip › biomolecules-2714095-supplementary.pdf]

Supplementary Materials

to

Physics of Ice Nucleation and Antinucleation: Action of Ice-Binding Proteins

Bogdan S. Melnik [1,*], Ksenia A. Glukhova [1], Evgeniya A. Sokolova (Voronova) [2,3], Irina V. Balalaeva [2], Sergiy O. Garbuzynskiy [1] and Alexei V. Finkelstein [1, 4,5,*]

[1] Institute of Protein Research, Russian Academy of Sciences, 142290 Pushchino, Russia; gkseniya@gmail.com (K.A.G.); sergey@phys.protres.ru (S.O.G.)
[2] Institute of Biology and Biomedicine, Lobachevsky State University of Nizhny Novgorod, 603022 Nizhny Novgorod, Russia; malehanova@mail.ru (E.A.S.); irin-b@mail.ru (I.V.B.)
[3] Pushchino Scientific Center for Biological Research, Institute of Cell Biophysics, Russian Academy of Sciences, 142290 Pushchino, Russia
[4] Faculty of Biotechnology, Lomonosov Moscow State University, 142290 Pushchino, Russia
[5] Faculty of Biology, Lomonosov Moscow State University, 119192 Moscow, Russia
* Correspondence: bmelnik@phys.protres.ru (B.S.M.); afinkel@vega.protres.ru (A.V.F.)

Genetic constructs

To express mIBP83-GFP fusion protein in mammalian cells, mIBP83 fragment of pEX-A2-SBP-T plasmid was subcloned into pTagGFP2-N vector (Evrogen) by PCR with the primers
5′ ATTCTCGAGATGAGCGTGACCAACACCAAC 3′;
5′ ATAGGATCCCCCACGCCGCTAATTTTCAC 3′.

pEX-A2-SBP-T plasmid was purchased from Eurofins Genomics (Germany).

To produce mIBP83 protein in *E.coli* cells, DNA fragment encoding mIBP83 was subcloned into pET22b vector (Novagen) by restriction digestion of pEX-A2-SBP-T with NdeI and SalI.

To produce mIBP83-GFP fusion protein in *E. coli* cells, DNA fragment encoding mIBP83-GFP was generated by overlapping extension PCR using pEX-A2-SBP-T and pGFP-cycle3 [1] with the following primers:
T7Prom 5′ CCCGCGAAATTAATACGACTCACTAT 3′;
mIBP83r 5′ GCCGCTCGCGCCCACGCCG 3′;
GFPf 5′ GCGAGCGGCACCGGCATGGCTAGC 3′;
T7Ter 5′ CTAGTTATTGCTCAGCGGTGGC 3′.

The amplified mIBP83-GFP fragment was inserted into pET28a vector (Novagen).

The plasmid DNA for the RmAFP1 protein was constructed from an amino acid sequence taken from the Protein Data Bank structure 6XNR, which represents AFP1 isoform antifreeze protein from the longhorn beetle, *Rhagium mordax*.

For all the constructs, the correct reading frames were confirmed by DNA sequencing.

Molecular weight of RmAFP1 protein is 12.8 kDa.

Molecular weight of mIBP83 protein is 8.3 kDa. mIBP83 is a mutant protein previously constructed by us based on *Choristoneura fumiferana* antifreeze protein isoform 337 (Uniprot accession number Q9GTP0_CHOFU). The design of this mutant protein is described in detail in the previous work [2]. In short, nine point mutations (C4V, C17V, C67A, C80V, I68T, T66G, T49K, C62V, and C85V) were made, to minimize a probability of non-native disulfide bridges formation during the protein isolation and purification as well as to optimize the protein surface for binding to ice. In addition, Y26 is in the mutant instead of 3,5-diiodotyrosine in the wild-type protein. At last, N- and C-termini were slightly truncated (by two and by five residues, correspondingly) compared to the wild-type protein.

Molecular weight of mIBP83-GFP fusion protein is 34.6 kDa.

Isolation and purification of mIBP83, RmAFP1 and mIBP83-GFP proteins

E. coli BL21 (DE3) strain was used for expression of the proteins. Transformation of competent *E. coli* cells was conducted using pET-22b plasmid encoding the target protein. Cells were cultivated in liquid LB medium of the following composition (per 1 L): trypton, 10 g; yeast extract, 5 g; NaCl, 10 g; NaOH (10 M), 600 μl. Kanamycin was added to the growth medium as a selection marker in 1:100 ratio, the gene of resistance to this

antibiotic was encoded in the target vector plasmid. To stimulate the yield of the target protein, IPTG was added to the cultural medium to 100 mM final concentration. High-pressure homogenizer was used for cell disintegration. All the solutions were prepared using de-ionized and distilled water.

Protein isolation from *E. coli* cell lysate was conducted by sequential gel filtration, ion-exchange and hydrophobic chromatography. The purity of protein preparations was analyzed by SDS-PAGE in 12% gel. Concentration of ice-binding protein mIBP83 was determined by ultraviolet absorption at 280 nm, taking its extinction coefficient (0.683) into account. Concentration of RmAFP1 protein was determined by ultraviolet absorption at 215 nm and 225 nm by method of Waddell [3]. Concentration of GFP and mIBP83-GFP proteins was determined at 395 nm wavelength corresponding to absorption band of the chromophore group of the green fluorescent protein.

As GFP, cycle3-GFP variant of green fluorescent protein was used in all the visualization experiments. It will be named simply GFP. Cycle3-GFP folds well at +37 ^{o}C during expression in *E. coli* cells, that is why it is used in many studies as a "wild-type protein". Besides that, it is very important fot the goals of the current work that this protein does not dimerize at low concentrations (up to 2 mg/ml) [4, 5]. This was tested by sedimentation and analytical chromatography. mIBP83-GFP fusion protein was also analyzed by gel chromatography. The protein, as well as GFP alone, had an elution profile with one quite sharp peak, giving evidence that this protein does not dimerize.

The purity of protein preparations was analyzed by SDS-PAGE (Figure S1):

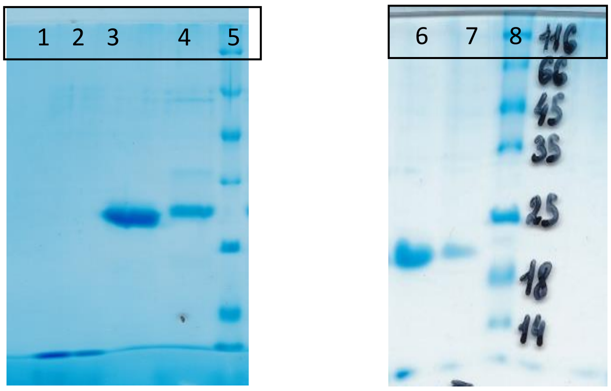

Figure S1. SDS-PAGE of the final stage of purification of proteins. The left gel is for mIBP83 protein, the right one is for RmAFP1 protein. Lanes 5 and 8 are markers of molecular weights, kDa. Lanes 1,2 are mIBP83; lane 3 is GFP; 4 is mIBP83-GFP fusion protein; 6,7 are RmAFP1.

The presented SDS-PAGE gels (Figure S1) show high purity of each of the obtained proteins (mIBP83, GFP, mIBP83-GFP, and RmAFP1).

Examination of the ability of mIBP83-GFP protein to bind to ice surface

To test the ice-binding ability of mIBP83-GFP fusion protein, the following experiment was carried out. Two identical tubes were filled with buffer solution (1.0 ml, 20 mM sodium phosphate buffer, pH = 7.0) and were frozen at -20 ^{o}C, then incubated at room temperature till the beginning of ice melting. Thus, each vial had a piece of ice surrounded by a small volume of liquid. Then, mIBP83-GFP solution was added to one tube, and GFP solution was added to another one (200 µl, 2 mg/ml). The tubes were irradiated by a transilluminator. If mIBP83 as a part of the chimeric protein would not lose its ability to bind to ice surface, it should cover the piece of ice in the tube, and the piece should fluoresce more intensely than the solution. GFP lacks the ability to bind to ice, so it would not be seen on the ice piece. Figures S2 and S3 show the photographs of such experiments.

It can be seen in Figure S2 that the main fluorescing component in the tubes with mIBP83-GFP was the piece of ice. In tubes with GFP alone (without mIBP83, see Figure S3), the solution, but not ice, fluoresce (mainly in the bottom, because the tubes were irradiated from below).

Thus, one can conclude that the protein mIBP83 attached to GFP did not lose the ability to bind to ice surface.

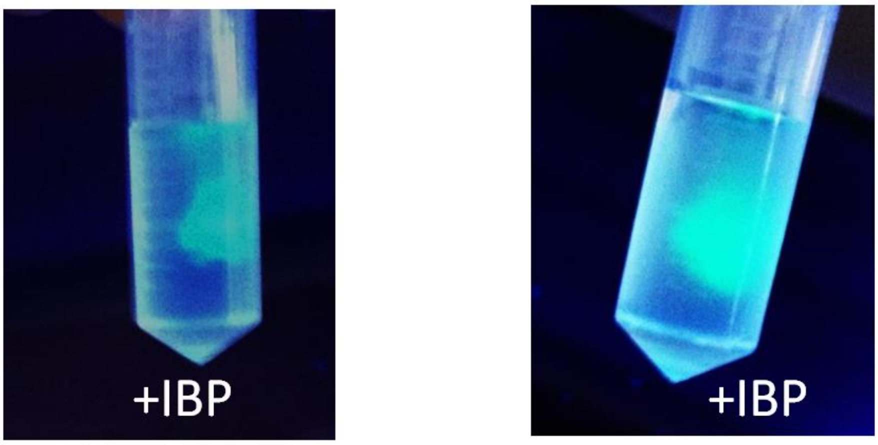

Figure S2. Binding of mIBP83-GFP to ice. A piece of ice in a test tube with mIBP83-GFP solution. The tubes were irradiated with ultraviolet light from below, a transilluminator was used for this purpose. Mostly ice glows.

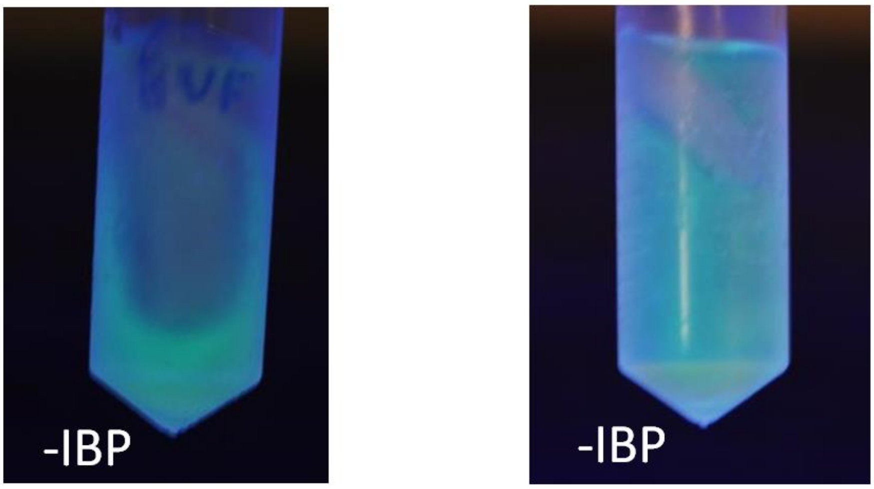

Figure S3. A piece of ice in a test tube with GFP solution. Same as in Figure S2, the tubes were irradiated with ultraviolet light from below, with the same transilluminator. Mostly the solution glows, not ice.

References

1. Melnik, T.N.; Povarnitsyna, T.V.; Glukhov, A.S.; Melnik, B.S. Multi-state proteins: approach allowing experimental determination of the formation order of structure elements in the green fluorescent protein. *PLoS One* **2012**, *7*, e48604. doi: 10.1371/journal.pone.0048604
2. Deeva, A.A.; Glukhova, K.A.; Isoyan, L.S.; Okulova, Y.D.; Uversky, V.N.; Melnik, B.S. Design and analysis of a mutant form of the ice-binding protein from *Choristoneura fumiferana*. *Protein J.* **2022**, *41*, 304–314. doi:10.1007/s10930-022-10049-6
3. Waddell, W.J. A simple ultraviolet spectrophotometric method for the determination of protein. *J. Lab. Clin. Med.* **1956**, 48, 311–314.
4. Fukuda, H.; Arai, M.; Kuwajima, K. Folding of green fluorescent protein and the cycle3 mutant. *Biochemistry* **2000**, *39*, 12025–12032. doi:10.1021/bi000543l
5. Glukhova, K.F.; Marchenkov, V.V.; Melnik, T.N.; Melnik, B.S. Isoforms of green fluorescent protein differ from each other in solvent molecules "trapped" inside this protein. *J. Biomol. Struct. Dyn.* **2017**, *35*, 1215–1225. doi:10.1080/07391102.2016.1174737
